# Spatial Distribution of Flagellated Microalgae *Chlamydomonas reinhardtii* in a Quasi-Two-Dimensional Space

**DOI:** 10.3390/mi14040813

**Published:** 2023-04-02

**Authors:** Tetsuo Aono, Kyohei Yamashita, Masafumi Hashimoto, Yuji Ishikawa, Kentaro Aizawa, Eiji Tokunaga

**Affiliations:** Department of Physics, Faculty of Science, Tokyo University of Science, 1-3 Kagurazaka, Shinjuku-ku, Tokyo 162-8601, Japan

**Keywords:** microswimmer, spatial distribution, variance-to-mean ratio, microalga, *Chlamydomonas*, shim ring, flagella, Poisson distribution, binomial distribution, hydrodynamic interaction

## Abstract

Although the phenomenon of collective order formation by cell–cell interactions in motile cells, microswimmers, has been a topic of interest, most studies have been conducted under conditions of high cell density, where the space occupancy of a cell population relative to the space size ϕ>0.1 (ϕ is the area fraction). We experimentally determined the spatial distribution (SD) of the flagellated unicellular green alga *Chlamydomonas reinhardtii* at a low cell density (ϕ≈0.01) in a quasi-two-dimensional (thickness equal to cell diameter) restricted space and used the variance-to-mean ratio to investigate the deviation from the random distribution of cells, that is, do cells tend to cluster together or avoid each other? The experimental SD is consistent with that obtained by Monte Carlo simulation, in which only the excluded volume effect (EV effect) due to the finite size of cells is taken into account, indicating that there is no interaction between cells other than the EV effect at a low cell density of ϕ≈0.01. A simple method for fabricating a quasi-two-dimensional space using shim rings was also proposed.

## 1. Introduction

The spatial distribution (SD) of organisms in ecology and of photons in a propagating light beam in quantum optics [1] are important research targets, respectively, for understanding the influence of the competition between organisms and their environment on survival and the quantum optical properties of photon populations. It is well known that, when there is no correlation between individual organisms or photons and the distribution is completely random, the distribution becomes a Poisson distribution (PD) [2] and the variance is equal to the mean. On the other hand, if there is an interaction between individuals, i.e., in the case of swarming (attractive) interaction, the variance > mean (bunching, super-Poisson), and in the case of avoiding (repulsive) interaction, the variance < mean (anti-bunching, sub-Poisson) [3]. Therefore, the ratio of variance/mean is a good indicator to extract the characteristics of inter-individual interactions from the distribution. Even at the scale of microorganisms, i.e., cells, emergent behavior such as the ordered structure of cell populations created by intercell interactions is a major field of research [4] from the viewpoint of the origin of multicellularity in biology and from the interest in the relationship with statistical phase transition phenomena in physics. This is also classified as a field of active matter. In particular, while there is a wealth of theoretical studies on the formation of population structure by way of the hydrodynamic interaction of flagellated unicellular microorganisms (called self-propelled particles or microswimmers in the field of active matter), experimental verification is less abundant compared to theoretical studies. In particular, to the best of our knowledge, we can find no examples in which the SD of motile cells has been examined using the variance/mean (V/M) ratio index. In this paper, we experimentally investigated the SD in the quasi-two-dimensional (quasi-2D) space of *Chlamydomonas*, a unicellular green alga with flagellar motility, to explore cell–cell interactions using the V/M ratio.

The SD and pattern formation of individual organisms has long been the subject of much study in ecology. It is well known that fish schools and birds flock together to form certain spatial patterns [5,6,7], and it has been argued that these patterns are formed as hydrodynamically stable (energy-saving) arrangements [8] and are also advantageous to individual survival by reducing the risk of predation [9,10,11]. On the other hand, it has been reported that trees in forests avoid each other in order to share sunlight and soil nutrients [12]. In terms of distribution patterns, the former can be classified as a concentrated (clumped) distribution and the latter as a uniform distribution, as opposed to a random distribution with no interaction (directly or indirectly) between individuals.

In *Chlamydomonas* and *Euglena*, unicellular organisms with flagellate locomotion, the interaction of phototaxis and convection results in crowding and the formation of spatially non-uniform patterns, known as bioconvection [13,14]. While some believe that bioconvection is beneficial to cells, i.e., due to a better supply of oxygen (non-photosynthetic cells), a better supply of nutrients (in all cases), or equal opportunity to receive light (photosynthetic cells), there are reports of experiments that do not support significant positive effects of bioconvection [15].

The collective patterning of unicellular organisms is also an important issue in the search for the origin of multicellularity. For example, slime molds usually live in small, unicellular amoebae but, when exposed to starvation, they aggregate in large numbers to form fruiting bodies [16,17]. Algae of the Volvocales, which are thought to have become multicellular by the unicellular green alga *Chlamydomonas* forming colonies, are being studied as a model organism for elucidating the origin of multicellularity [18].

The collective spatial pattern formation mentioned above is also in a field of active matter, and there have been detailed experimental studies and theoretical analyses of the collective motion and pattern formation of micromachines, i.e., micrometer-sized self-propelled particles [19]. The fundamental question is how can collective patterns be created when the overall spatial pattern is not visible to the individual particles and the particles have no intention of creating it. If the individuals are inanimate particles, it must be understood purely in terms of physical interactions between particles, and in the case of biological cells, in addition to physical interactions (hydrodynamic interactions in flagellated unicellular organisms [20]), the effects of attraction or avoidance by chemicals such as metabolites can also be a factor [17,21].

Besides bioconvection, collective behavior due to hydrodynamic interactions between flagellated microorganisms is well known [4]. There are a considerable number of theoretical and experimental studies on the transition of motile cell populations to clustered or phase-separated states in three-dimensional or quasi-2D space depending on cell number density or volume fraction, since the swimmer–swimmer interaction is a complicated function of their relative displacement and orientation [22]. Such a transition occurs at dense populations well above ϕ=0.1, where ϕ is the area fraction [4,23]. In this study, by contrast, we investigated the sparse density region of ϕ≪0.1 that has not received much attention, where there is seemingly no apparent cell clustering or ordered state in the cell population. Note that the maximum density reached by the growth of *Chlamydomonas reinhardtii* (IAM C-541) in a normal culture environment is $1.0\times 10^{7}$/mL (stationary phase) [24]. Its volume fraction is ϕ=0.005. In other words, the upper limit of natural density for cells is about ϕ=0.01. Inter-cell interactions up to this density have not been investigated.

The purpose of this paper is to determine whether cell–cell interactions are attractive or repulsive in flagellated *C. reinhardtii*, which is also a model organism for exploring the origin of multicellularity, in its normal culture condition. To this end, we experimentally examined the SD of cells to determine whether cells tend to cluster or avoid each other during locomotion. In this case, it is necessary to suppress the formation of SD by the bioconvection of cells. Therefore, the space was restricted to be a quasi-2D space perpendicular to the direction of gravity and the direction of light (of uniform intensity) irradiation, so that the cells would not be affected by gravity and phototaxis when viewed from the cells. Although various microdevices have been created in the study of microorganisms in quasi-2D space [21,25,26,27], the present study also proposes an inexpensive and simple method for creating 2D space by using commercially available SUS304 shim rings. Although aggregation due to oxygen gradients has been reported [21] regarding the behavior of relatively high-density *C. reinhardtii* populations in a quasi-2D space, the characteristics of the SD of cells in the absence of explicit aggregation at low density have not been elucidated, as pointed out above. In this study, we measured the 2D distribution precisely by capturing moving images with a camera, analyzed the distribution pattern using a partitioning method, and compared it with the results of Monte Carlo simulation (MCS). The results showed that the distribution was almost consistent with the random distribution, considering the excluded volume effect (EV effect) due to the finite size of the cells, and it could be concluded that there was no inter-cell interaction other than the EV effect.

## 2. Basis of Spatial Distribution of Cells

In this paper, VMR= variance-to-mean ratio (V/M ratio) was used as a measure to characterize the SD. As is well known, VMR=1 if individuals are placed in space completely randomly without interaction with each other, resulting in a PD. If individuals avoid each other, the distribution tends from random to uniform (sub PD) with VMR<1 and, if they crowd together, the distribution tends to be clumped (super PD) with VMR>1. In reality, cells of finite size were placed in a quasi-2D restricted space of finite size and thickness, so the analysis was performed taking into account that there may be deviations from the PD even if the cells were placed randomly. The following three points were considered in handling the experimental results.

(1) Cells occupy a finite space equal to their size and cannot occupy the same space multiple times. This is called the EV effect, or steric interaction, in which cells avoid each other, making *VMR* smaller. The larger the space occupied by a cell, the more pronounced this effect becomes.
(2) If the space is not uniform and there is inhomogeneity, the distribution is spatially biased, which is an environmental factor that increases VMR. Therefore, it was necessary to carefully check for this effect.
(3) The purpose of this study is to determine whether interactions between cells, except for the excluded volume effect, tend to be attractive or repulsive.

The mathematical model of the SD is summarized as follows [28].

The 2D SD of cells considering the excluded volume (excluded area) effect can be approximated by the following model of a discrete (⇔continuous) probability distribution. When n=45 cells of area A=1 are placed in a space of area S=900, the probability of x cells entering a subspace of area D=100 can be approximated by the hypergeometric distribution as follows. The 2D space can be regarded as an assembly of neatly arranged M(=S/A) seats without a gap in this space. One seat is occupied by exactly one cell. The probability of x cells occupying the selected K(=D/A) seats is expressed by the hypergeometric distribution,

(1) $$P\left(x\right)=\frac{{}_{K}C_{x}\cdot {}_{M-K}C_{n-x}}{{}_{M}C_{n}}=\frac{{}_{100}C_{x}\cdot {}_{900-100}C_{45-x}}{{}_{900}C_{45}}.$$

Here, the number of sections of space =N=S/D=M/K=9, the probability of a cell entering in the subspace D within S is p=D/S=1/N, and the area ratio of cells to space (cell occupancy) is nA/S=0.05. The mean and variance of the hypergeometric distribution and their ratio are

(2) $$mean=n\frac{K}{M}=45\times \frac{100}{900}=5,$$

(3) $$variance=n\frac{K}{M}\left(1-\frac{K}{M}\right)\frac{M-n}{M-1}=45\frac{100}{900}\left(1-\frac{100}{900}\right)\frac{900-45}{900-1}=4.227,$$

and

(4) $$VMR=n\frac{K}{M}\left(1-\frac{K}{M}\right)\frac{M-n}{M-1}/n\frac{K}{M}=\left(1-\frac{K}{M}\right)\frac{M-n}{M-1}=\left(1-\frac{1}{N}\right)\frac{M-n}{M-1}.$$

The SD in the absence of EV effects can be obtained here by taking the limit of cell area A→0, i.e., M→∞, while keeping D/S=K/M=p=1/N constant and, as is well known, in this limit, the hypergeometric distribution becomes the binomial distribution,

(5) $$P\left(x\right)={}_{n}C_{x}\cdot p^{x}\cdot {\left(1-p\right)}^{n-x}={}_{45}C_{x}\cdot {\left(\frac{1}{9}\right)}^{x}\cdot {\left(1-\frac{1}{9}\right)}^{45-x}.$$

The mean and variance of the binomial distribution and their ratio are

(6) $$mean=np=45\times \frac{1}{9}=5$$

(7) $$variance=np\left(1-p\right)=4.44$$

and

(8) $$VMR=1-p=1-\frac{1}{N}.$$

As is well known, taking the limit n→∞ and p=D/S→0 as np=const=λ here, the PD,

(9) $$P(x)=\frac{e^{-\lambda }\lambda^{x}}{x!},$$

is obtained. The mean and variance of the PD and their ratio are

(10) $$mean=np=45\times \frac{1}{9}=5,$$

(11) variance=np=5,

and

(12) VMR=1.

To realize this situation experimentally, both the number of cells n and the area of the space S must be multiplied by c times, and the limit of c→∞ must be taken. Since the experiment was conducted in a finite space, it was not a PD, and the V/M ratio VMR depended on the number of sections N.

The approximation of the distribution with EV effect by the hypergeometric distribution is considered reasonable when the cell occupancy is sufficiently smaller than 1, but the closer the occupancy approaches to 1, the worse the approximation becomes. The reason is that the maximum value of n is M=S/A, which means that 100% cell occupancy is possible in the hypergeometric distribution that the cell centers are assumed to be placed in on the neatly spaced grid points in a discrete manner. However, in reality, the maximum occupancy is considered to be significantly smaller than 100% because cell centers are randomly placed at any position in the continuous coordinates of space. Therefore, an MCS as described below was used to theoretically predict the distribution with the EV effect. In Figure S1 of Supplementary Material (SM), it is shown that there is a remarkable difference at ϕ=0.1 and a detectable difference even at ϕ=0.01 in the V/M ratio between a hypergeometric distribution and that by MCS with the EV effect.

## 3. Experimental Methods

### 3.1. Sample Preparation

The wild type *Chlamydomonas reinhardtii* (IAM C-541) was used as a sample. *C. reinhardtii* grows by photosynthesis and swims in a breaststroke-type manner with two flagella. The body is nearly spherical, about 10 µm in diameter, senses light at the eyespot, and moves to the optimal light environment for photosynthesis. The average forward speed of *C. reinhardtii* is about 100 μm/s [29]. *C. reinhardtii* was cultured in TAP (Tris–Acetate–Phosphate) medium for 7 days. Cells were kept aerobically under continuous illumination (white LED light: 10 $\mu mol{\;m}^{-2}\cdot s^{-1}$) and at a constant temperature (23 °C).

### 3.2. Preparation of Quasi-2D Spatial Device

**Figure 1:**
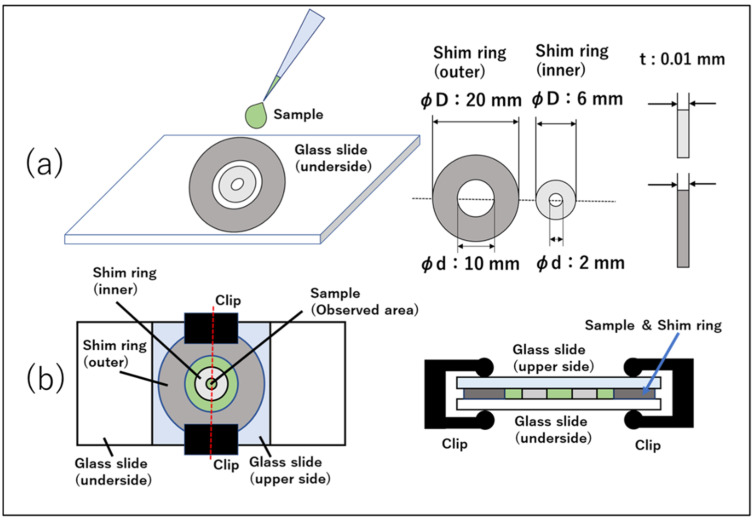
Method for fabricating quasi-2D microdevice. (**a**,**b**) Procedure for fabricating the device. Two rings of different sizes are used. Thickness tolerance is ±0.003 mm for both rings. (**b**) Top view and cross section from the side of the completed device. A clip is placed directly above the outer ring, which serves as a spacer.

*C. reinhardtii* was confined in a quasi-2D well with a diameter of 2 mm using a shim ring (SUS304, Iwata Corporation, Seki-shi, Gifu, Japan) with a thickness of 0.01 ± 0.003 mm. A shim ring with an inner diameter of 2 mm and an outer diameter of 6 mm was placed in the center of a ring with an inner diameter of 10 mm and an outer diameter of 20 mm on a glass slide (Figure 1a). Then, a cell suspension was dropped within the inner shim ring and covered with a glass slide. Clips were used to secure the two glass slides (Figure 1b). The outer ring was not relevant for observation but served as a spacer to prevent deflection of the glass for covering. It also prevented the inner ring from being directly exposed to air. This prevented air bubbles from entering the quasi-2D wells and enabled long-term observation.

### 3.3. Experimental Apparatus

**Figure 2:**
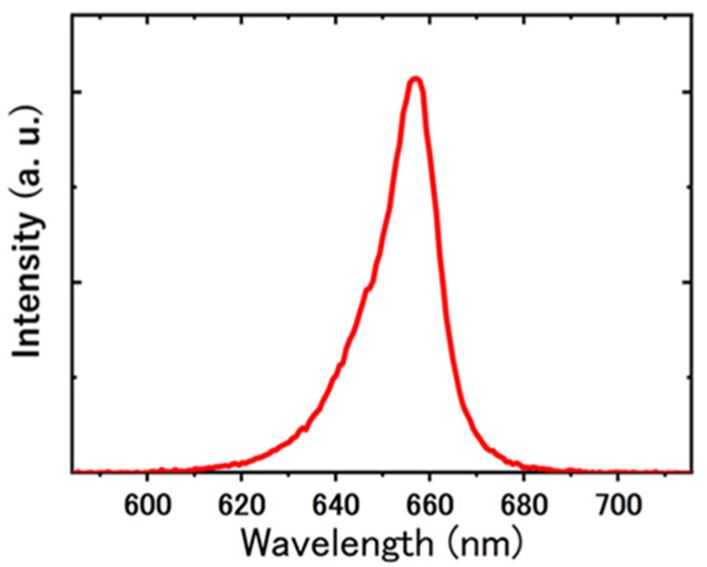
Luminescence spectrum of the red LED.

We used a digital biological microscope (GR-D8T2, Shodensha Inc., Osaka, Japan) fitted with a red LED (OSR5XNE3C1S, Xeon 3 Power Red Star LED, OptSupply, Hong Kong). An objective lens with a magnification of 10× (NA 0.25, Shodensha Inc., Osaka, Japan) was used. Red light was used for observation [30] to prevent *Chlamydomonas* from adhering to the glass. Experiments were conducted in the dark with light other than the observation light blocked. The center wavelength of the LED was about 660 nm. The beam diameter at the sample position was 5 mm and the photon flux density was 11.8 $\mu mol{\;m}^{-2}\cdot s^{-1}$. The emission spectrum of the red LED was measured with a miniature fiber optic spectrometer (USB2000, OptoSirius Corporation, Tokyo, Japan) and its spectral sensitivity was corrected with a standard light source (LS-1 Tungsten Halogen Light Source, Ocean Insight, Orlando, USA). The result is shown in Figure 2. A microscope camera (MC500W-G1-D, TAMAPACK, Osaka, Japan) with a 10× magnification eyepiece (CHU30-C-RS, Shodensha Inc., Osaka, Japan) was mounted on the microscope and the video image was recorded at a frame rate of 30 fps and a size of 2592 × 1944 pixels (1.53 × 1.15 $mm^{2}$).

### 3.4. Image Analysis

Recently, a review of methods for counting microorganisms using microscopic images has been reported [31]. The 2D SD of the cells was obtained by cell counting using ImageJ software on still images extracted from the video images, using one of the counting methods explained in that review, as follows.

#### 3.4.1. Frame Interval

The data were obtained by analyzing 15 min of the video image in a single analysis. However, if the number of frames was too large, it was impossible to analyze the data within the limited memory of the PC. Therefore, frames that were multiples of 30 in the raw data were extracted and imported into ImageJ. As a result, a total of 900 frames at 1 fps for 15 min were analyzed. Although the thinning of the video may cause tracking failures in rare cases, it does not seem to affect the results because the coordinates for the center of each cell necessary for this study were output without problems. Figure S2 of SM shows that analysis at different frame rates with different thinning rates makes little difference in the distribution. Figure S3 shows that a 1-s interval between analyzed frames is adequate to assure the independence of the spatial distribution of each frame.

#### 3.4.2. Binarization and Particle Tracking

A central 1944 × 1944 px^2^ (1.15 × 1.15 mm^2^) square section of the output 2592 × 1944 px^2^ (1.53 × 1.15 mm^2^) size was used for analysis to avoid the effects of micro-well boundaries. These movies were binarized by ImageJ [32]. In order to remove salt and pepper noise, a 4.1 × 4.1 $\mu m^{2}$ median filter was used. The background luminance was also subtracted. Particle tracking was performed on the binarized image using the ImageJ plug-in “particle tracker”. The obtained center-of-cell coordinates were output as a csv file.

#### 3.4.3. Cell Counting

A Python program was used to count the number of cells from the coordinates of the resulting csv file. The square observation area was divided into N(=m×m) sections, and the number of cells within each section was counted. Cells whose centers were on the boundary line were counted as being in the right or upper compartments. A histogram of the cell distribution was created from the counting data obtained, and the mean and variance values were calculated.

### 3.5. Calculation Method for Simulating the Spatial Distribution

(1) Following the procedures (2) and (3) below, n circular cells of diameter d were distributed in a square 2-dimensional space of length a on one side. The diameter d was assumed to be the same for all the cells. The programming language used was Python.
(2) The coordinates of the geometrical center of the cells were determined. First, a set of two-dimensional real random numbers between 0 and 1 was generated by the Monte Carlo method. Next, this random number was multiplied by the side length of the square area for observation, a=1944 px (1.15 mm), to give each cell a random coordinate within the observation area. In addition, taking into account the EV effect of *C. reinhardtii*, the distance between two coordinates should not be smaller than the diameter d= 16.9 px (10 μm). That is, for the *i*-th and *j*-th coordinates,
  $$\sqrt{{\left(x_{i}-x_{j}\right)}^{2}+{\left(y_{i}-y_{j}\right)}^{2}}\geq d.$$
  The cell of coordinates that did not satisfy this condition were not added, and the coordinates were again determined from random numbers.
(3) The 2D space was divided into N=m×m sections equally and the number of cells in each section were counted.
(4) (2) and (3) were regarded as one frame and repeated 900 times to obtain the same number of frames as in the experiment.
(5) A histogram was created with the horizontal axis as the number of cells in a section and with the vertical axis as the frequency of the occurrence of the section containing that number of cells.

Possible program errors were checked as follows. We confirmed that the V/M ratio is the theoretical value VM≈np(1−p)=(n/N)×(1−1/N) for the binomial distribution when the diameter d is set to 0 and that Kullback–Leibler divergence ≈ 0, which represents the difference between the simulated SD and binomial distribution models.

### 3.6. Simulation including Stationary Cells

Simulations were performed to examine the effect of stationary cells (SCs) on SD. Because some cells temporarily slow down their swimming due to collisions or changes in direction, we defined SCs as cells that continued to move at a speed of less than 18 µm/s per frame for more than 30 s. Their coordinates were output and placed as SCs in the simulation space. After that, cells were placed with randomly generated coordinates until the average number of cells observed in the experiment was reached. The method used was the same as in Section 3.5, and the EV effect of the SC was also taken into account.

## 4. Results and Discussion

### 4.1. Change in the Number of Cells over Time

**Figure 3:**
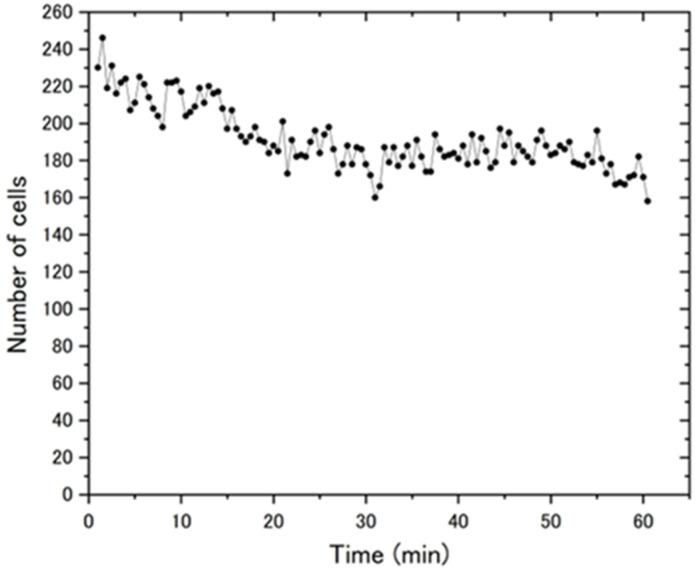
Change over time in the number of cells within the observation area.

*C. reinhardtii* was enclosed in a pseudo-2D space with a shim ring of 10 μm thickness and 2 mm inner diameter. The observation area was set at the center of the ring, and a decrease in the number of cells in the area was observed immediately after encapsulation (Figure 3). When the sample was first set up, the SD of the cells was often uneven, and cells flowed into and out of the observation region in the center of the ring immediately after encapsulation. It took about 20 min for the number of cells in the observation area to become almost constant (=steady state). Images after 20 min were used in the analysis. As shown in Figure S4, the time to reach a steady state depends on the sample, and 20 min is a conservative estimate.

At one frame every 30 s, particles in the observation area (1944 × 1944 px^2^, 1.15 × 1.15 $mm^{2}$) were counted using a particle tracker and the number of particles in each frame was plotted.

### 4.2. Partitioning and Cell Counting

**Figure 4:**
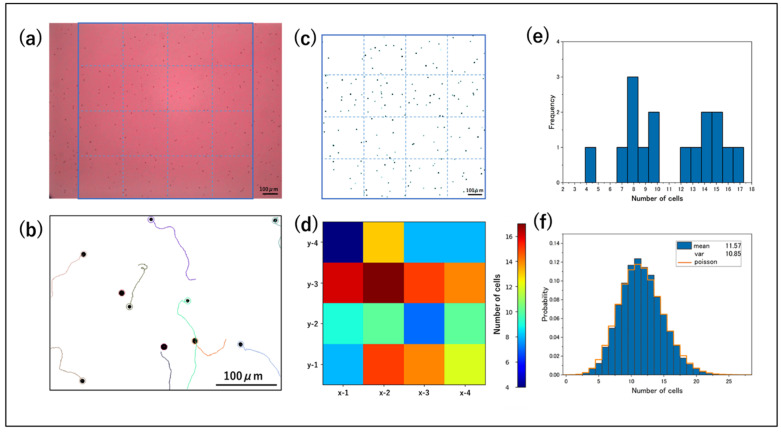
Analysis using 4×4 partitioning and particle track. (**a**) Acquired videos and the observation area used for analysis (in blue box). The background appears red because the red LED (660 ± 10 nm) was used for observation. The stage of the microscope was adjusted so that the center of the ring was at the center of the movie. The square area in the center was used for analysis. (**b**) Particle tracking for 2 s at 30 fps. The trajectory of the tracked particles is shown. The trajectory is not output for a stationary object. (**c**) Partition in the binarized image. (**d**) Heatmap of counts in (**c**). (**e**) Histogram at 1 frame in (**c**). (**f**) Histogram over 900 frames for 15 min. A video adjusted to be one frame per second (by thinning out 29 frames) was analyzed.

A red LED (660 ± 10 nm) was used as the light source for observing the square in the center of the observation area (Figure 4a). It has been reported that flagella of *C. reinhardtii* adhered to the glass can be detached by red light [30]; however, it is difficult to completely eliminate adhesion, and a certain number of SC are included (Figure 4b). By setting an upper speed limit for particle tracking, only SC can be tracked. The coordinates of the observed SC were then used in the simulation. That is, the SC were placed at the same coordinates as the experimentally observed ones, and then the cells that could move were randomly placed. Binarized movies were used for particle tracking (Figure 4c). A median filter was used in the binarization process to eliminate small glass contaminants and debris, and a heat map was made by counting the number of cells in a section in the frame shown in Figure 4c,d. The sections with high cell counts were colored red and those with low cell counts were colored blue. A histogram of only the frame in Figure 4c is shown in Figure 4e.

### 4.3. Experimental Results and Cell Distribution in Simulation

**Figure 5:**
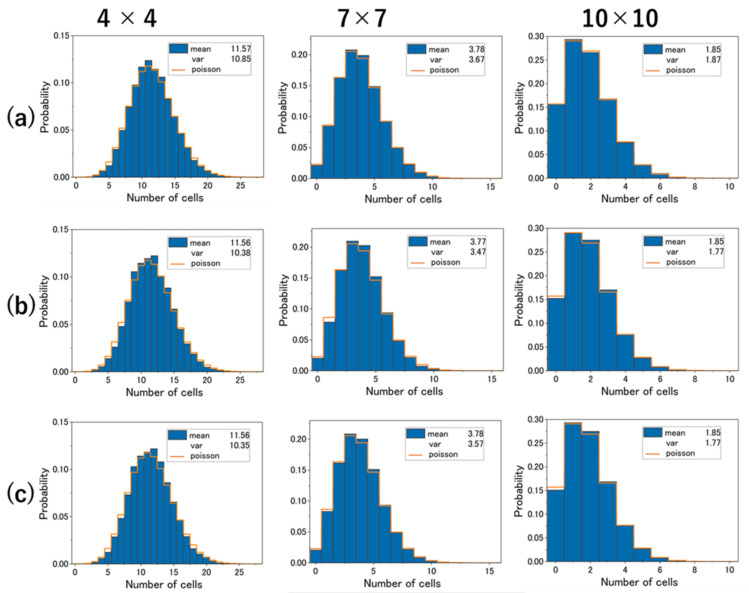
Histograms in m×m (m=4,7,10). (**a**) Histograms in the experimental results. The period between 35 and 50 min from the beginning of the observation was analyzed. The video used for the analysis was thinned to 1 fps, and the total number of frames was 900. The average number of cells was 185.18 per frame. (**b**) Cell distribution by MCS with observed SC placed. The simulation was performed with a total number of 185 cells in each frame, which was the sum of the SC and the cells at randomly generated coordinates. The diameter of the cells was set to 10 µm and they were arranged so that they did not overlap. This was performed from frame 1 to frame 900. (**c**) The coordinates of all 185 cells were generated randomly. As mentioned above, the EV effect was also taken into account. This was repeated 900 times to generate a histogram.

There is a so-called EV effect, in which space is occupied depending on the area of the individual. The simulation was performed with the cell size set to 10 µm in diameter and no overlap allowed within that range. In reality, there is a variation in *C. reinhardtii* cell size, but this was not taken into account in this simulation. Generally, when coordinates are randomly generated, an ideal point distribution is obtained and can be approximated by a binomial distribution, in which case the V/M ratio is 1−1/N when the number of sections is N. However, in the simulation in which the coordinates of cells are randomly generated (Figure 5c), the V/M ratio is smaller than 1−1/N. This is due to the fact that the simulation includes the EV effect, which causes substantial avoidance interactions. In the experiment (Figure 5a), however, the variance is larger than in the simulation with all the cells randomly placed (Figure 5c). This is also true for simulations that include SC (Figure 5b). In other words, in the experiment, the SC occurred in specific sections, imposing environmental effects such that the distribution was locally crowded.

### 4.4. V/M Ratio vs. Number of Sections

**Figure 6:**
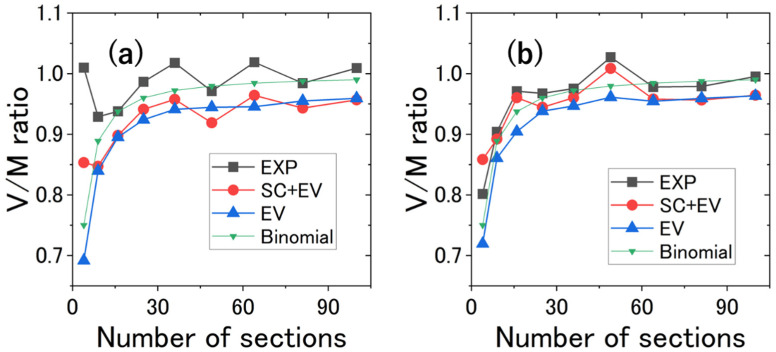
The V/M ratio as a function of the number of sections. The experimental results (EXP), the simulation results with both stationary cells (SC) and excluded volume (EV) effect considered, and those with only the EV effect. For reference, VMR for the binomial distribution in Equation (8) is displayed. VMR for the hypergeometric distribution in Equation (4) is evaluated to be nearly (~99%) the same as that for the binomial distribution. (**a**) The average number of cells per frame was 185.18; the average number density per $mm^{2}$ was 139.95. Assuming that each cell is a circle with a diameter of 10 µm, the area fractions of all the cells in 2D space was 1.1%. The volume fraction in 3D space was 0.76% when each cell was assumed to be a sphere with a diameter of 10 µm. The same data as in Figure 3 were used. (**b**) The average number of cells was 104.66. The average number density per mm^2^ was 80.04 and the area fraction was 0.6%. The volume fraction was 0.41%.

The V/M ratio was plotted as a function of the number of sections N from 2×2 to 10×10 in Figure 6. Since no interactions other than the EV effect were taken into account in the simulation, any other interactions would be expected to affect the value of the variance. The experimental variance was larger than that of the simulation with all the cells randomly placed. At first glance, this seems to be a crowding effect. It should be noticed, however, that the dependence of the variance on the number of sections was reproduced fairly well by the simulation including the effect of SC.

For reference, part of the movie from which the cell distribution data in Figure 6a was extracted is given as Video S1 in the SM.

It is known that the size of the measurement area can affect the results of spatial pattern analysis depending on the cluster size of the cells [33]. In this study, the V/M ratio shows characteristic dependence on the size of the section being measured. Note also that this feature was reproduced by the simulation that includes SC. In this simulation, coordinates other than those of the SC were randomly generated and interactions such as the hydrodynamic effects of the swimming cells were not taken into account. Therefore, the spatial pattern is considered to be formed by SC.

As shown in Figure S5 of the SM, an apparently spatially-biased distribution increases the variance. Thus, extrinsic environmental effects (EE) that bring spatial inhomogeneity, such as those listed below, increase the variance more than random placement. In addition to spatial inhomogeneities such as the distribution of SC, the existence of boundaries, non-uniform irradiation light intensity, non-uniform thickness in quasi-2D space, and temporal variations in the number of cells in the observed region also cause increased variance in the present experimental method, which extracted images from a series of time-varying movies. The reason why the boundary wall of the shim ring was not included in the observation area was to eliminate the existence of the boundary and to make the space for cells as uniform as possible, but this did not necessarily mean that the number of cells in the observation area was preserved. Therefore, as described in Figure 3, still images were extracted from movies in time periods when the number of cells was almost constant.

What all of the above means is that, despite the best experimental efforts to satisfy the condition of spatial uniformity, there can be EE of uncontrollable inhomogeneity, so there must always be pressure to bias the cells toward crowding. Conversely, the only effect that biases them toward avoiding each other is intrinsic, endogenous cell-to-cell interactions (CI, both avoiding and crowding interactions are possible, which is exactly what we are interested in in this study), other than the excluded volume (EV) effect.

Figure S6 in SM shows eleven observations in addition to the two in Figure 6. In Table S1, the experimental conditions for the results in Figure S6 are tabulated. In all thirteen dependences of the V/M ratios on the number of sections in Figure 6 and Figure S6, the magnitude of the V/M ratio is in the order of experiment (CI + EV + SC + EE) ≳ simulation (EV + SC) ≳ simulation (EV). 

Eleven of the thirteen observations clearly show experiment (CI + EV + SC + EE) > simulation (EV + SC), and the remaining two (g and i in Figure S6) show experiment (CI + EV + SC + EE) ~ simulation (EV + SC), with none showing experiment (CI + EV + SC + EE) < simulation (EV + SC). From this, it can be reasonably interpreted that, in the former case, uncontrolled inhomogeneous environmental effects were introduced, while in the latter case, uniform environmental conditions were met, which led to this result. In other words, there is no endogenous cell–cell interaction (CI) in the experiment, thus experiment (EV + SC + EE) ≳ simulation (EV + SC): ~ or > is determined by whether the experiment eliminated (~) or did not eliminate (>) environmental effects that are difficult to control. If there exist cell–cell interactions, then for crowding interactions it should always be experiment > simulation regardless of spatial inhomogeneity, and for avoiding interactions there should be a case where experiment < simulation when the condition of no environmental effects is met. Therefore, we can conclude that no cell–cell interaction other than the EV effect is exerted at ϕ~0.01 in quasi-2D space.

In Figure S7 of the SM, the V/M ratio is analyzed for the number of sections N greater than 100. Note that the V/M ratio converges to a constant value as N is increased for both experiment and simulation, and that the converged value is less sensitive to stationary cells. This is expected from the N dependence of VMR theoretically predicted in Section 2. When we focus on the V/M ratio for the largest N in Figure 6 and Figure S6, the above conclusions are further supported. At N=100, the experimental V/M ratios are the same or smaller than VMR for the binomial distribution for eight observations.

In Figure S8 of the SM, the radial distribution functions of the SD for Figure 6b were calculated. One can see that the experimental SD does not reflect 100% of the EV effect due to the size distribution of cells, which is not considered in the simulation, and inevitable errors in determining cell coordinates in the experiment. The above conclusions are further reinforced when these effects, which increase the V/M ratio of the experiment in addition to the environmental effect, are also taken into account.

## 5. Conclusions and Prospects

At a sparse cell density as small as ϕ≈0.01 (≈100 cells/$mm^{2}$, average intercell distance ≈ 100 µm) in quasi-2D space with the same vertical thickness as the cell diameter, swimming *Chlamydomonas* do not affect each other except in collisions. Recalling that the stationary-phase cell density of *Chlamydomonas* is ϕ=0.005, the absence of cell–cell interactions up to ϕ=0.01 may indicate that occupancy below this level is a comfortable environment for cells not to interfere with each other, which might be why ϕ=0.01 is a natural upper limit of cell density. The next interesting issue is where the interaction is initiated as ϕ is increased.

Certain *Chlamydomonas* species are edible and have been suggested to produce functional components [34]. It is also capable of generating hydrogen gas, a clean energy resource, via photosynthesis [35,36]. Knowing the cell–cell interactions of *Chlamydomonas* and predicting their SD will contribute to food and energy research by promoting a higher efficiency of bioreactors in biomass production technology by using them.

## Data Availability

The data presented in this study are available on request from the corresponding author.

## Supplementary Materials

The following supporting information can be downloaded at: https://www.mdpi.com/article/10.3390/mi14040813/s1. Figures S1–S8, Table S1, and Video S1 are available. Video S1 is a part of the original movie for the data in Figure 6a.
