# Supplementary material for: Spatial Distribution of Flagellated Microalgae Chlamydomonas reinhardtii in a Quasi-Two-Dimensional Space
Source: Micromachines (Basel). 2023 Apr 2;14(4):813. doi: 10.3390/mi14040813 (PMC10146014; doi:10.3390/mi14040813)
Supplement: Supplementary file 1 [file micromachines-14-00813-s001.zip › Supplementary Figures and Table.pdf]

Supplementary Materials

Spatial Distribution of Flagellated Microalgae *Chlamydomonas reinhardtii* in a Quasi-Two-Dimensional Space

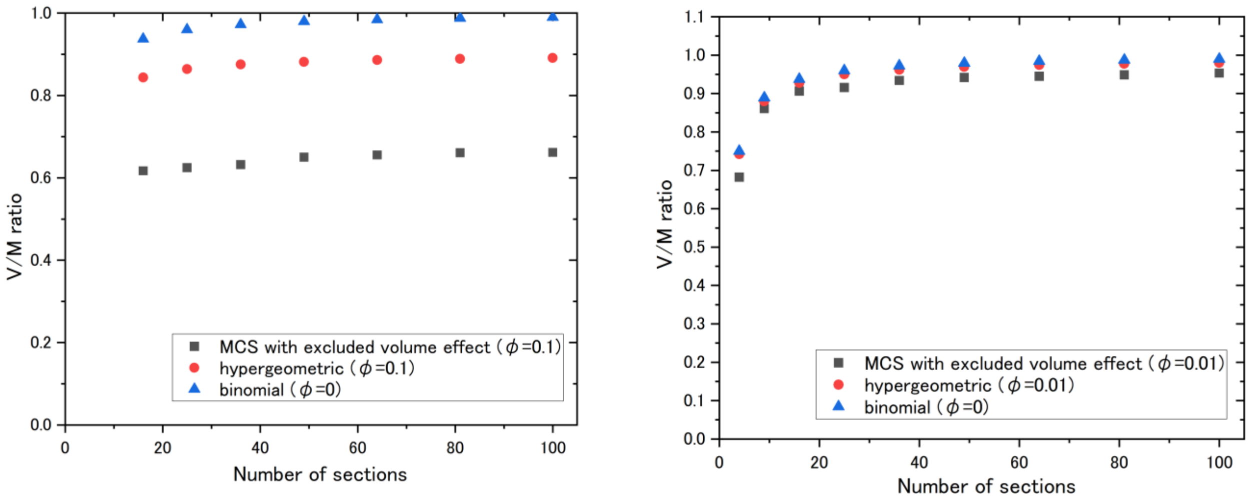

Figure S1. Dependence of the V/M ratio on the number of sections for three cellular spatial distribution (SD) models (binomial, hypergeometric, and Monte Carlo simulation) at high cell density (area fraction $\phi = 0.1$) and low cell density ($\phi = 0.01$).

Calculation method for the results in Figure S1

(1) MCS (Monte Carlo simulation)

Cells were randomly placed in a two-dimensional space on the conditions as follows:

Cell diameter: d =16.9 px (10 μm)

Observation area: $S = 1944 \times 1944$ px^2 (1.15 $\times$ 1.15 mm^2)

Total number of frames: 900

Area fraction: $\varphi = \frac{\pi\left(\frac{d}{2}\right)^2 n}{S}$ (d: diameter of cells, n: total number of cells, S: observation area)

$\phi = 0.1$ when the total number of cells is 1685.

$\phi = 0.01$ when the total number of cells is 169.

(2) V/M ratio of hypergeometric distribution: $VMR = \left(1 - \frac{1}{N}\right)\frac{M-n}{M-1}$, where $M = \frac{S}{\pi\left(\frac{d}{2}\right)^2}$ and $n = M\phi$.

(3) V/M ratio of binomial distribution: $VMR = 1 - \frac{1}{N}$

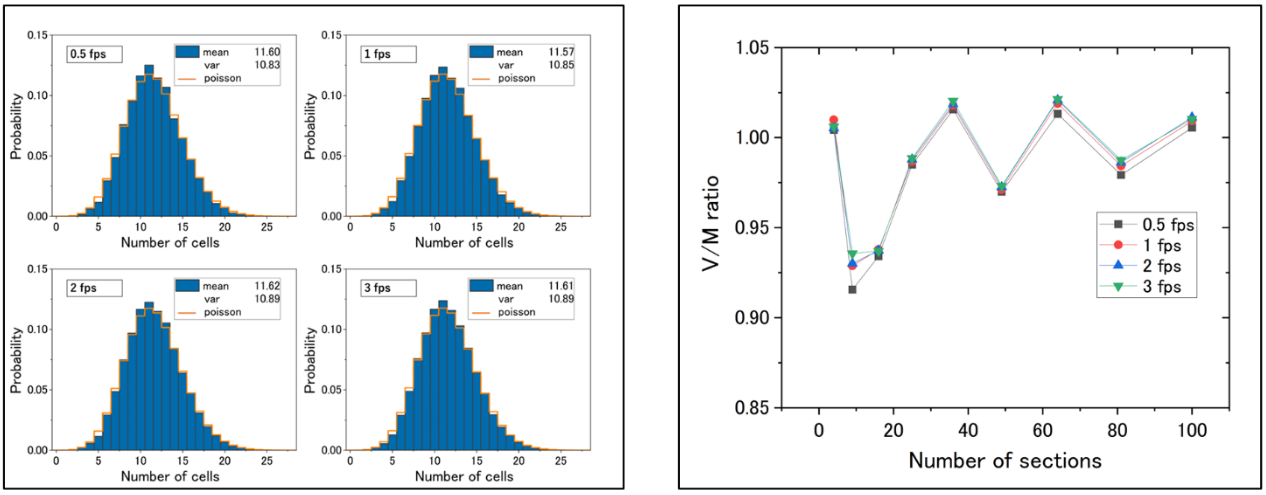

Figure S2. Analysis at different frame rates with different thinning rates. Analysis with the same data as in Figure 5 in Main text. Histograms are counts when the observation area is divided into $N = 4 \times 4$.

When considering random point placement, there should be no correlation between the previous and following frames. However, it is impossible to create a perfectly correlation-free video due to the problem of still individuals and other factors. Therefore, the number of frames per second was reduced by subtracting the frames between frames in the video acquired at 30 fps. The histograms and V/M ratios compared between 0.5 and 3 fps are shown in the figure. The frame rate did not significantly affect the results.

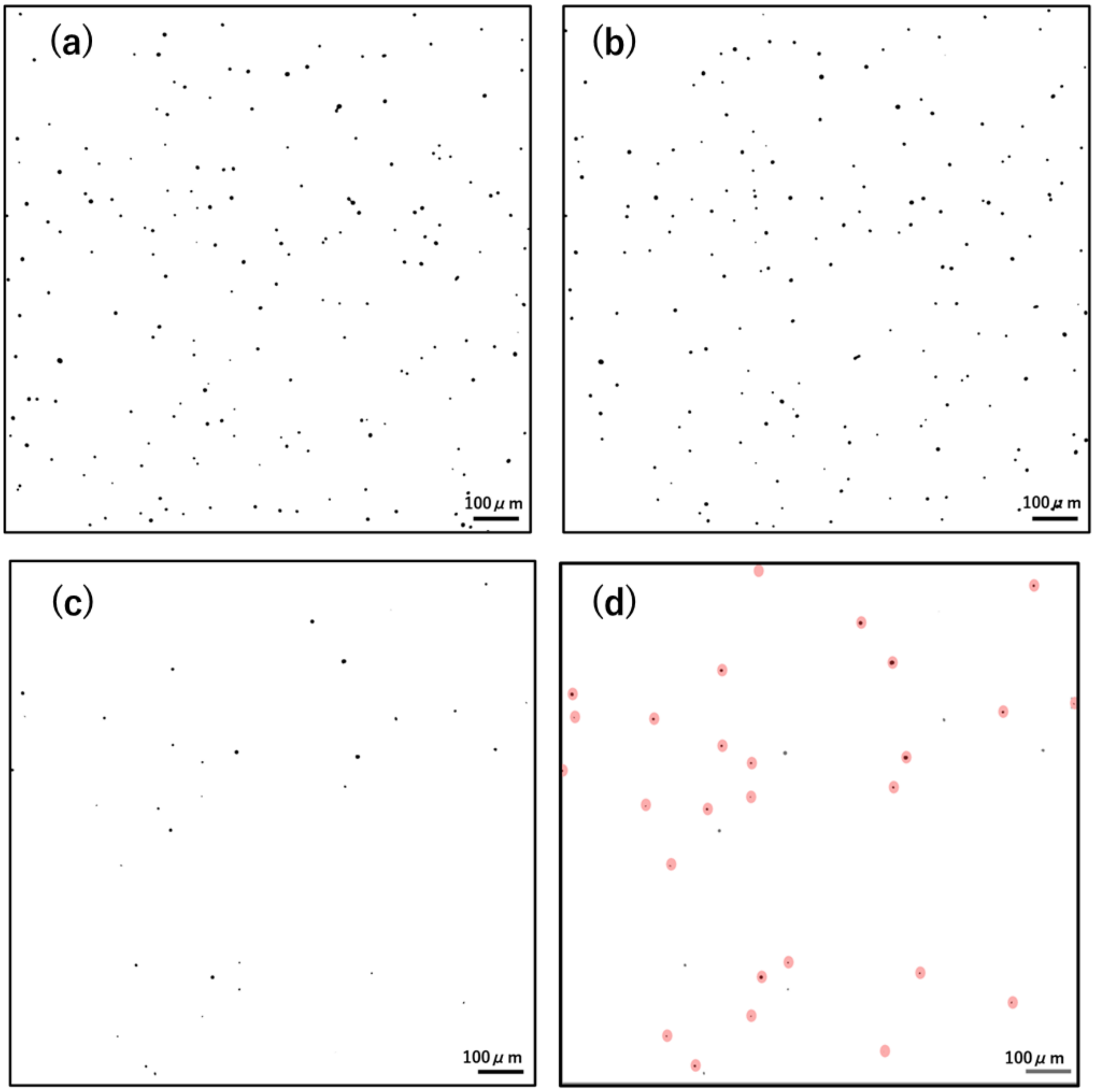

Figure S3.

(a) Image of the first frame of the video used for analysis.

(b) Image of the next frame of (a). One second passed from the frame (a).

(c) Result of image (a) multiplied by image (b).

(d) Coordinates of stationary cells were plotted over (c).

The SD of the experiment is obtained by summing the distributions of frames extracted at regular intervals from the video. To obtain the reliable experimental SD, it is required that the SD of each frame is statistically independent. The video was shot at 30 fps, and the frames were thinned out to 1 fps for analysis. Although frames (a) and (b) in Figure S3 are 1 second apart, the coordinates of cells remaining after multiplying these images (=coordinates of cells that have not moved during 1 second) almost coincide with those of stationary cells (=cells that have not moved for more than 30 seconds). This suggests that there is little correlation between swimming cells in the 1 fps movie and that independence of distribution

is assured.

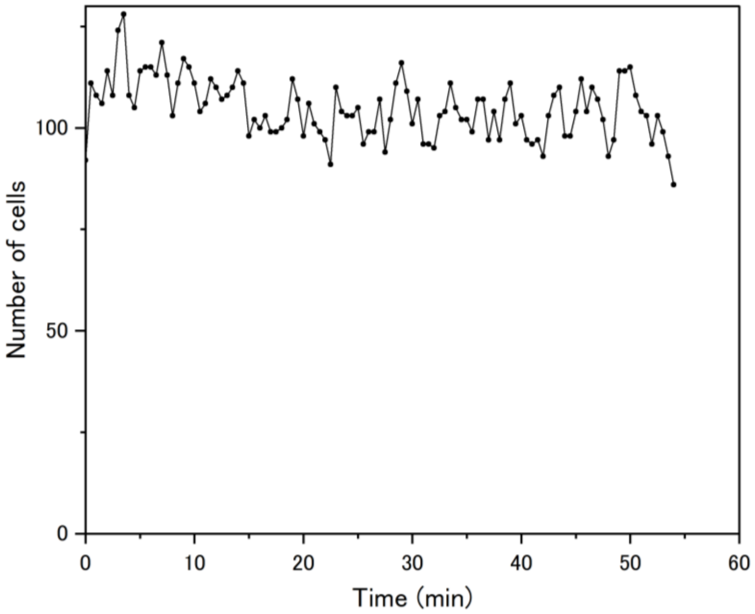

Figure S4. Change over time in the cell count from the time of cell inclusion in the two-dimensional well of the sample in Figure 6(b)

The time required to reach a steady state depends on the initial position and number of cells at the time of cell inclusion. Compared with Figure 3, the cell count is almost stable before 20 minutes in Figure S4. Twenty minutes to reach a steady state is a conservative estimate.

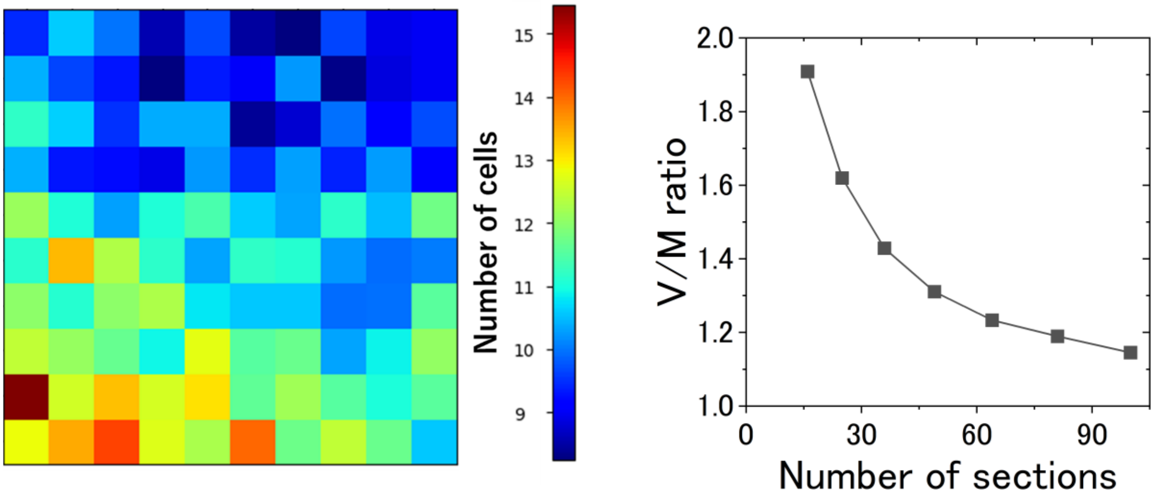

Figure S5. V/M ratio and heat map for overtly biased cell SD. A shim ring with an inner diameter of 3 mm and a thickness of 20 μm is used. White LED with light intensity of 0.56 μmol $m^{-2} \cdot s^{-1}$. Average number of cells is 1078.58 and volume fraction is 2.13%.

The variance is clearly larger when analyzing a sample with an apparent non-uniform SD. The reason for such a distribution is not clear, but the possible causes are as follows. The beam diameter was 4 mm, but the light intensity may have been non-uniform due to the larger inner diameter of the shim ring, in which case the distribution may be non-uniform due to the phototaxis of *C. reinhardtii*. Another possible factor could be non-equilibrium at the time of encapsulation. In some of the data shown in Figure 5, the variance was clearly larger than expected. This phenomenon may be caused by environmental factors and needs to be verified.

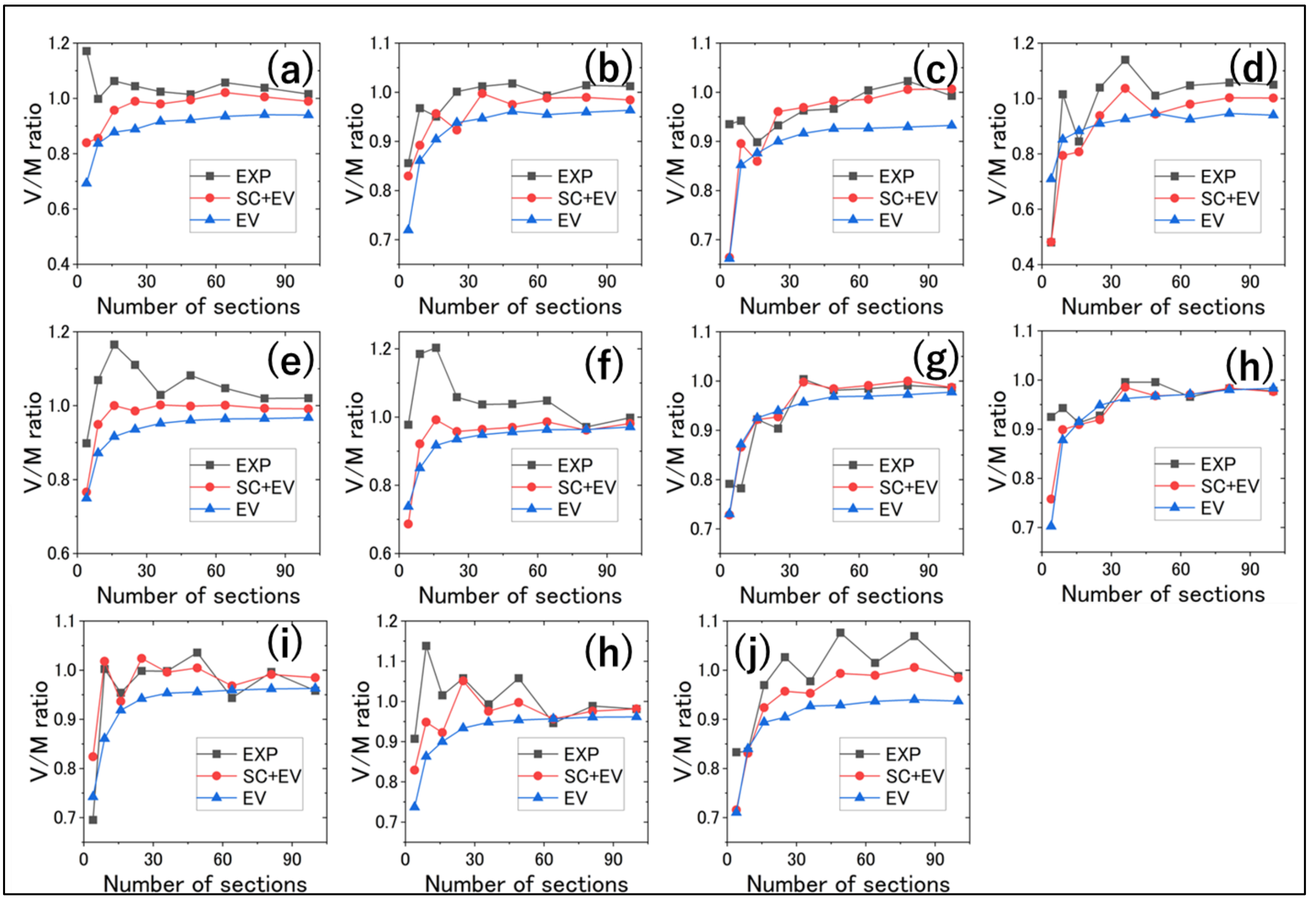

Figure S6. Relation between V/M ratio and number of sections.

The experimental results (black square, EXP), the simulation results with both stationary cells (SC) and excluded volume (EV) effect, and those with only the EV effect.

Table S1. Experimental conditions for the results in Figure S6 (a)-(k).

	Thickness of the space (mm)	Photon flux density (μmol · m^{-2} · s^{-1})	Average number of cells per frame	Volume fraction (%)	fps	Note
a	0.01	11.8	185.87	0.74	1	Different time period for

						the same sample as in Figure 6a.
b	0.01	11.8	105.32	0.41	1	Different time period for the same sample as in Figure 6b.
c	0.01	11.8	258.65	1.02	1	Different time period for the same sample as in d.
d	0.01	11.8	250.78	0.99	1	
e	0.02	27	372.94	0.74	3	Different time period for the same sample as in f.
f	0.02	27	364.75	0.72	3	
g	0.02	11.8	219.03	0.43	3	Different time period for the same sample as in Figure i.
h	0.02	11.8	211.24	0.42	3	
i	0.01	0.59	120.62	0.48	3	Different time period for the same sample as in j.
j	0.01	0.59	120.44	0.48	3	
k	0.01	0.59	246.32	0.97	3	

Figure S6 shows the relation between the SM ratio and the number of sections for each of the experimental conditions listed in Table S1. Simulations were performed assuming an excluded volume effect that allowed half of the cell size (5 μm) to overlap in the 20 μm restricted space.

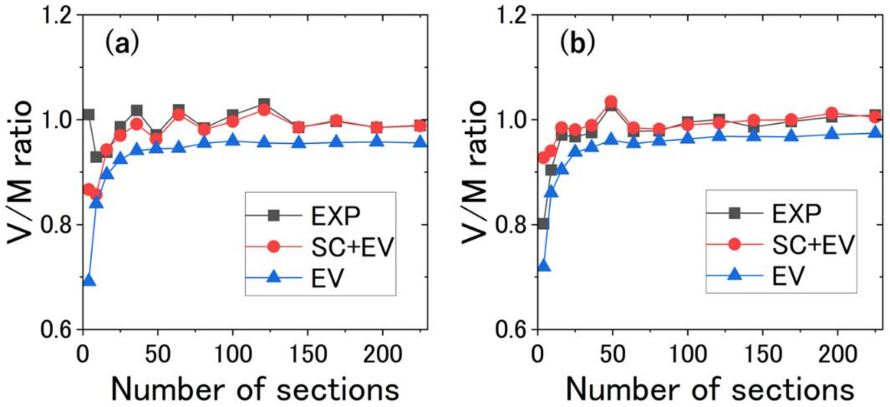

Figure S7. (a) Dependence of the V/M ratio on the number of sections N up to 225 for the same experimental data as in Figure 6a. (b) Dependence of the V/M ratio on the number of sections N up to 225 for the same experimental data as in Figure 6b. EXP and SC+EV agree well for larger N. It is suggested that the V/M ratio converges to a constant value as N is increased, but the converged value is influenced by stationary cells.

As shown in Figure S7, the V/M ratio converges to a constant value as the number of sections N is increased for both experiment and simulation. This is expected from theoretical prediction as discussed in Sec. 2.

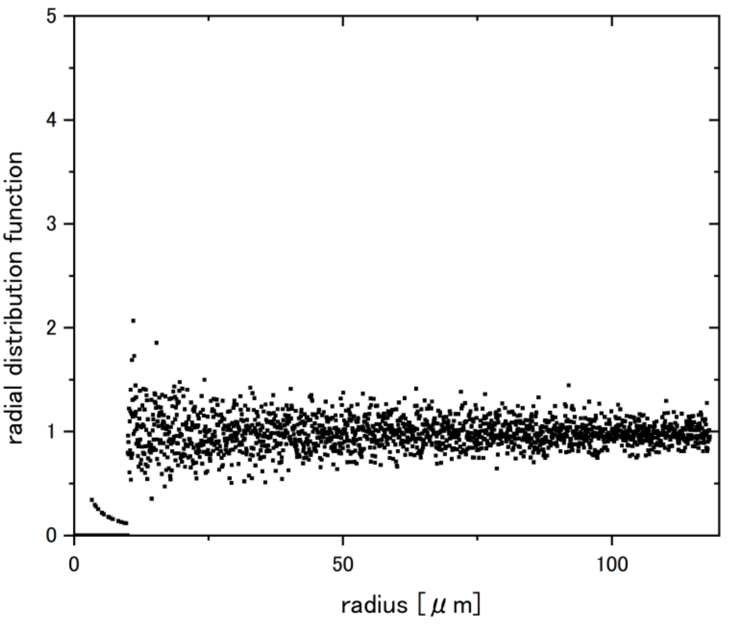

Figure S8. Radial distribution function of the SD simulation data (EV) in Figure 6b.

As a supplementary analysis, the radial distribution function (RDF) of the SD by Monte Carlo simulation was calculated as shown in Figure S8, while the RDF of the experimental SD was not properly obtained because of the difficulty in accurately determining the center of cells that are not neat circles in the following procedure.

Analytical Procedure for Calculating Radial Distribution Function from Spatial Distribution of particles

The RDF for the SD of particles is defined as follows.
$n(r)$ = the number of particles within a ring of radius r and thickness Δr from the particle of interest
$2\pi r\Delta r\rho$ = the number of particles expected to fall within the same ring (ρ = population density of the system [/px^2]).
The RDF for the particle of interest is obtained by $f(r) = n(r)/2\pi r\Delta r\rho$.
The RDF for the SD is obtained as the particle and frame averages of $f(r)$.

The radius was set in 0.1 increments from 0.1~200 pixel. Here, the maximum radius was taken to be at least 10 times larger than the diameter of the cell (16.9 pixel = 10μm).

For each frame, we selected cells whose maximum radius does not exceed the observation range, and counted the number of cells whose center fell within each ring. The number of cells counted was then averaged over the cell and frames. This was divided by $2\pi r \Delta r \rho$ to obtain the RDF.

Video S1. A part of the original movie for the data in Figure 6a.
